# Enhancing Patient‐Centred Care and Cultural Safety in Medical Imaging: The Radiographers Experience of Communicating With Patients in a Multicultural and Multilingual Setting in Auckland

**DOI:** 10.1002/jmrs.70005

**Published:** 2025-07-10

**Authors:** Nica Abrasado, Sibusiso Mdletshe

**Affiliations:** ^1^ Department of Anatomy and Medical Imaging, Faculty of Medical and Health Sciences The University of Auckland Auckland New Zealand; ^2^ Radiology Department Te Whatu Ora Counties Manukau (Middlemore Hospital) Auckland New Zealand

**Keywords:** communication, cultural competence, cultural diversity, culturally competent care, person‐centered care

## Abstract

**Introduction:**

Effective communication between patients and healthcare professionals has been shown to contribute to beneficial patient outcomes but requires recognition of linguistic and cultural differences. This is critical in a locality like Auckland which has been shown to be the most diverse region in New Zealand in terms of ethnicity, languages and culture. English is the most spoken language in New Zealand, followed by Te Reo Māori. The aim of this qualitative, phenomenological study was to explore and describe the experience of radiographers communicating with patients in a multi‐cultural, multi‐lingual healthcare setting in Auckland, New Zealand.

**Methods:**

The research study population included radiographers registered with the Medical Radiation Technologists Board (MRTB) employed at the study location. The participant recruitment process included convenience, purposive, and snowball sampling. Data was collected through individual interviews that were audio‐recorded and transcribed verbatim, with the sample size (*n = 11*) determined through the saturation of themes. Data was analysed by means of Tesch's framework for data analysis.

**Results:**

Four themes emerged: (a) cross‐cultural challenges in patient communication; (b) enhanced patient‐centred care through culturally responsive communication; (c) tailored communication methods based on contextual patient factors; and (d) adaptive communication strategies.

**Conclusion:**

This study underscores the importance of adaptive communication in overcoming linguistic and cultural barriers, emphasising the need for culturally safe and patient‐centered care while maintaining professionals' responsibility to provide quality care to diverse patient populations.

The findings have relevance beyond Māori context, highlighting the changing role of radiographers towards equitable and culturally sensitive healthcare.

## Graphical abstract

This study underscores the importance of adaptive communication in overcoming linguistic and cultural barriers, emphasising the need for culturally safe and patient‐centred care while maintaining professionals' responsibility to provide quality care to diverse patient populations. The findings have relevance beyond the Māori (New Zealand indigenous people) context, highlighting the changing role of MITs towards equitable and culturally sensitive healthcare.

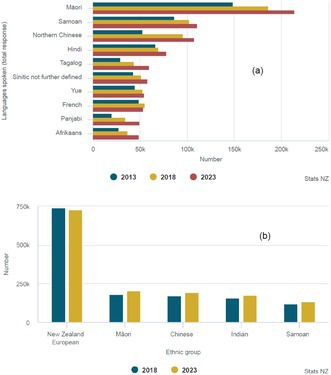

*It is much more important to know what sort of patient has a disease than what sort of disease a patient has*. William Osler.

## 1 Introduction

Medical imaging (MI) practice requires effective patient communication, coupled with empathy and a focus on patient‐centred care, ultimately contributing to patients' diagnoses and appropriate treatment management [1]. Effective communication contributes to good patient outcomes but requires recognition of linguistic and cultural differences [1, 2]. Healthcare professionals who feel ill‐prepared for cross‐cultural encounters may harbour fears of appearing discriminatory or displaying inappropriate behaviour, which could lead to a widened communication gap with patients [1, 3]. This could impact both cultural competence and cultural safety practice. Cultural competence entails understanding and responsiveness to various cultural aspects such as race, gender, sexual orientation, social class, and economic factors within culturally diverse communities [1, 3, 4]. In contrast, culturally safe practice requires continuous critical reflection on power differentials and recognising existing interpersonal power dynamics between healthcare providers and patients. It demands the acquisition of essential skills, knowledge and attitudes to ensure the delivery of secure, accessible, and responsive healthcare, particularly for indigenous communities, but has the potential to benefit all patients [5, 6].

Developing appropriate communication methods within a multicultural environment gives rise to language barriers and exposes healthcare professionals to cultural values, attitudes and beliefs that differ from their own [1, 3]. Failing to acknowledge and respect these differences can lead to miscommunication that, in the context of MI, disconnects the radiographers from each patient's needs. There is a predetermined expectation that radiographers are educationally prepared and assume adequate professionalism to provide culturally sensitive and patient‐centred care without fault. This expectation stems from radiographers inherent role as a patient advocate and their nurturing responsibilities [3, 7]. On the contrary, the limitations of building rapport with the patient and obtaining diagnostic quality images within the time constraint of a single radiographic procedure can compromise optimal, patient‐centred care [3].

**FIGURE 1:**
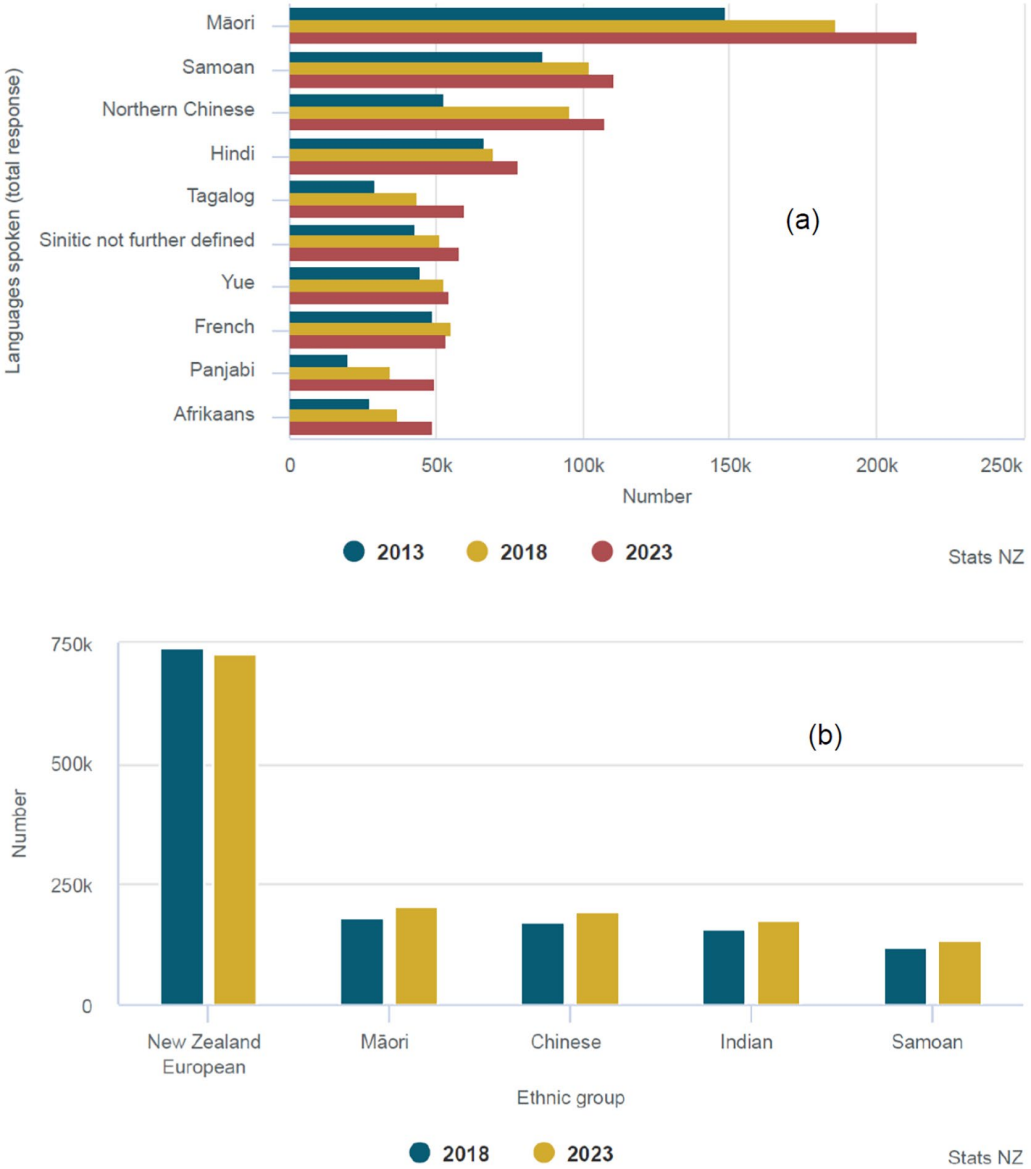
(a) Ten most widely spoken languages in 2023 (excluding English), for census usually resident population count, 2013–2023 Censuses, and (b) Number of people in the five largest ethnic groups in Auckland region, for census usually resident population count (level 3 ethnic group), 2018–2023 Censuses [8].

In New Zealand, while English is the most widely spoken language, several other languages are spoken by over 50,000 people, Māori (indigenous people's language) being the most spoken language after English (Figure 1a) [8]. The other common official language (with about 25,000) is the New Zealand sign language which has about 25,000 users [8]. The Auckland region has been shown to be the most diverse region in terms of ethnicity and would, therefore, be diverse in terms of languages (Figure 1b) [8].

While international research has explored the experiences of nurses [9, 10, 11, 12], student radiographers [13, 14], medical practitioners [15, 16] and patients' perspectives, [17, 18, 19] there is a notable gap when it comes to understanding radiographers unique experiences in a multicultural healthcare environment, especially in the New Zealand context.

This study, therefore, sought to answer the question ‘What are the experiences of radiographers communicating with patients in a multi‐lingual, multicultural healthcare setting?’ The aim of this study was to explore and describe the experiences of radiographers communicating with patients in a multicultural, multilingual healthcare environment in Auckland.

## 2 Methods

### 2.1 Study Design

This study employed a descriptive phenomenological qualitative design [20]. This approach was chosen to provide deeper insight into the experiences of radiographers employed at the study location while interacting and communicating with patients from diverse cultural and linguistic backgrounds. The research design was applied in two phases:

- Phase 1 explored the radiographers experience in communicating with patients in a multicultural environment in Auckland.
- Phase 2 focused on the development of guidelines. However, this phase will not be discussed in this article.

### 2.2 Population and Sampling

Non‐probability sampling (including convenience, snowball, and purposive) sampling methods were used to invite information‐rich participants. The study population included all the radiographers employed at Counties Manukau | Middlemore Hospital (single site) radiology department. The eligible study population consisted of 51 radiographers, while the sample size consisted of 11 radiographers, representing a range of experience levels and ethnicities, including one Māori participant. This sample size was selected based on the data saturation principle. The invitations were sent via email to some potential participants and posters were also distributed to different areas of the radiology department (i.e., main/inpatient X‐ray, emergency X‐ray and the outpatient clinic).

### 2.3 Data Collection

Data was collected from the beginning of June 2023 to the end of July 2023 through audio‐recorded, face‐to‐face interviews using a semi‐structured interview guide (Table 1). All interviews took place at the study location to ensure the environment was accessible and familiar. All interviews were meticulously audio‐recorded, ensuring an accurate and comprehensive record of each participant's interview duration. All interviews were conducted by a female fourth‐year honours student researcher who was attached to the study site as part of her training. The student was guided by the principal investigator (PI) who was an experienced African male radiographer who has conducted several qualitative studies and published them and had lived in New Zealand for more than 2 years and was not attached to the study location.

**TABLE 1:** Semi‐structured interview guide facilitating participant interviews.

Demographic information	Please give me details about your: – Name – Ethnicity – Years of experience as a radiographer
Broad opening statement	Tell me about your experience communicating with patients from different cultures and speaking different languages
Additional probing questions	Do you speak any other languages other than English? If so, how does this change your experience communicating with patients who also speak the language? Tell me about your experiences communicating with patients from different cultures/speaking different languages in the outpatient clinic compared to the emergency department. How do you adapt your communication style to make yourself understood? Can you give me specific examples that stood out to you? How do you feel when you encounter communication barriers? What would have helped you communicate more effectively in this situation? How have these language barriers affected the images you obtained during the examination (if at all)? Can you give me an example of a time when you communicated with patients of different cultures and spoke different languages where you learned something that you now apply in daily practice?

Each interview commenced with the following broad statement: ‘Tell me about your experience of communicating with patients from different cultures and speaking different languages’, additional probing questions were used to gather more specific information, as detailed in Table 1. After each interview, reflections were recorded by the interviewer as field notes, and the recording was shared with the PI. This process allowed for the identification of possible themes that were emerging, and interviews were conducted until data saturation was reached.

### 2.4 Data Analysis

The data analysis process followed Tesch's steps of qualitative data analysis, as outlined in Table 2. This systematic process was used to categorise the interview data into main themes and associated subthemes. The audio‐recorded interviews were transcribed verbatim and saved as Word documents, which were then uploaded onto a password‐protected project on NVivo 12, a qualitative data analysis software [22]. Each transcription was carefully read and reflected on to identify and code relevant quotes encompassing each participant's experience. Related codes were organised into general categories before rearranging and merging data into feasible themes [21].

**TABLE 2:** Tesch's systematic qualitative data analysis process [21].

Steps	Application to this specific study
Step 1. Get a sense of the whole	Audio‐recorded interviews were transcribed verbatim into interview transcripts, read, and then re‐read to extract meaning
Step 2. Pick one document (interview) to understand the “underlying meaning”	The researchers started by analysing the first interview conducted to understand the underlying meaning
Step 3. Make a list of topics emerging and cluster similar topics together	Main and supporting ideas were labelled using descriptive wording. Related topics were clustered together, resulting in the formation of a comprehensive list encompassing potential themes and subthemes
Step 4. Revisit data with topics, abbreviate the topics as codes	Data was correlated to the main themes/ideas. Each theme was assigned a code
Step 5. Turn topics into themes	Relevant verbatim quotations were assigned a code that correlated to the themes. Search for a pattern/link among the interview data was conducted. Comparisons were drawn to establish relationships and further condense themes/ideas
Step 6. Reduce the number of themes by grouping similar themes	Further organisation of coded data. Main themes and associated sub themes were renamed to coincide with the study aims and objectives
Step 7. Assemble the data material belonging to each category in one place and perform preliminary analysis	Data was grouped into respective categories. Preliminary data analysis was commenced
Step 8. If necessary recode your existing data	Established codes were analysed and saturated into identified main themes and associated subthemes

### 2.5 Trustworthiness

To establish the credibility and trustworthiness of this study, the researchers diligently followed and applied Guba's model [23]. The criteria for trustworthiness include dependability, transferability, credibility, and confirmability, which are essential facets of ensuring the quality of a qualitative research project. The researchers ensured confirmability by using triangulation and multiple data sources (observation, field notes) during the interviews to thoroughly understand the phenomenon [24]. Dependability was ensured through maintaining a comprehensive record of the research process and secure storage for future references at any stage after research completion [24]. Credibility of the study was achieved through triangulation and member checking. Transferability was achieved by provision of a comprehensive description of the research setting, data population, and methodology.

### 2.6 Ethical Considerations

The Auckland Health Research Ethics Committee (AHREC), University of Auckland, granted ethical approval of the study (reference number AH25471). The following principles were applied to ensure adherence to ethical conduct during the research process [25]:

- Respect for autonomy, right to equality, justice, human dignity and protection against harm [24, 26, 27].
- Right to privacy and confidentiality [28].
- Right to freedom of choice, expression, and access to information.
- Responsiveness to Māori—consultation with Māori was done with the Iwi United Engaged (IUE) [29], who supported the study.

### 2.7 Relevance to Māori

This study holds significance for Māori health as it has the potential to facilitate the ongoing reduction of health inequities among the Māori population. It addresses the role of suboptimal and ineffective healthcare communication, which may have contributed to the perpetuation of these disparities. The experiences of communication that the radiographers shared will be used to formulate communication guidelines to improve communication with patients and whānau (family), including Māori. Māori involvement and voice were ensured by purposefully inviting the radiographers who identified as Māori to participate.

## 3 Results and Discussion

Eleven interviews were conducted for this study and their demographics are summarised in Table 3. Their years of experience ranged from 6 months to 17 years.

**TABLE 3:** Participant demographics (*n* = 11).

Variable	Category	Number *n* = 11
Ethnicity	NZ European/Pākehā	4
	East/Southeast Asian	5
	South Asian	1
	Māori/NZ European	1
Gender	Female	9
	Male	2
Years of experience	0.5–1 year	5
	1.5–2 years	5
	10+ years	1

### 3.1 Themes

Data analysis yielded four themes reflecting the participants' experience of communicating with patients and how it affected their practice: (1) Cross‐cultural challenges in patient communication; (2) enhanced patient‐centred care through culturally responsive communication; (3) tailored communication methods based on contextual patient factors; and (4) adaptive communication strategies. The themes reflect that the concept of cultural diversity extends beyond one's ethnic or minority group within a social system. It encompasses the psychological dimensions of cultural diversity, including emotional states, beliefs and individual characteristics. It forms the basis for developing compassionate, patient‐centred care [1, 3, 30]. Further, patients tend to appreciate their experience within the healthcare system based on the established reciprocal, relational rapport more so than the technical aspects of acquiring diagnostic acceptable images [7, 31, 32].

### 3.2 Cross‐Cultural Challenges in Patient Communication

Brief introductions, adequate provision of examination instructions, and clear explanations of what to expect, facilitate a stronger patient‐radiographer relationship compared to brief, task‐focused imaging encounters [30, 31]. In the current study, participants recalled experiences where the patient generally felt confused and sometimes frustrated throughout the examination, and found it challenging to articulate the type of pain they experienced and follow positioning instructions due to communication breakdown.

I think they feel more confused, especially with, say you're doing a…like a rolled lateral, and you're like feeling on the other side, the patient may not be able to articulate that, oh, it's the left side, or it's the side…. They still don't understand what we're trying to say. [P1].

Sometimes, patients can get a bit frustrated when you can tell that they, they're trying to tell you something and they can't portray it to you… you know that they wanna tell you something, and you wanna understand it, but the communication just isn't there. [P5].

One participant qualified in CT explained the necessity for authorised consent in an immediate emergency, despite the patient's presenting linguistic barriers.

And like sometimes when there's just no way to get anyone to consent the patient in their language, then we just have to get a doctor to, like, do an authorised consent… so in those cases, the patient really doesn't know what is going on at all. [P8].

As a result, patients in these circumstances are unaware of the imaging examination altogether, and this could have unintended consequences as reported in other studies [33, 34].

Participants recalled a time when a Māori patient's previous experiences of personal disempowerment within the public healthcare system negatively influenced future encounters, including their interaction with the practitioners.

… They experienced a bit of racism… I was with another student and she was Māori as well, and we were able to talk to her and kind of help alleviate those, cause she didn't want to come to the hospital. Like she didn't show up for appointments a lot of the time because she didn't wanna experience the same thing. [P6].

Instances like these demonstrate the detrimental effect on patients on the receiving end of culturally insensitive communication in the healthcare context. It has been reported that Māori patients expressed dissatisfaction and hesitancy towards medical practitioners and nurses providing insufficient procedure explanations and treatments, often seeking additional support from extended family members, even when the family members lacked generalised knowledge of the information themselves [35]. The inclusion of family members or carers during examinations is often underutilised for optimising patient‐centred care, partly due to concerns about radiation safety [13]. While addressing these safety concerns is essential, evaluating the appropriateness of including family members or carers in the examination room contributes to the ongoing development of care practices within the medical imaging profession. Furthermore, including family members in the examination process aligns with the integral, invariable role of whānau (family) in fostering positive health outcomes in Māori culture [36]. Māori patients generally find that care received from whānau adequately addressed their wider taha wairua (spiritual health) and taha hinengaro (emotional health), which were often neglected in the mainstream healthcare system [35, 37].

On the contrary, adaptations in communication approaches extend beyond linguistic and cultural barriers, encompassing various patient presentations in the radiology department. Patients with intellectual disabilities, such as Down Syndrome, benefit significantly from pronounced facial expressions, intentional verbal and non‐verbal cues, variable tones and instructions in plain, simple language [38]. In the current study, the participants' perspectives offered valuable insight into the multifaceted challenges of linguistic and cultural barriers encountered during medical imaging examinations. These reflections reveal a heightened empathy among participants, particularly as they contemplate the often overwhelming feelings of unfamiliarity and anxiety that commonly confront patients upon entering the emergency X‐ray department.

I just feel for them even more, cause I'm like, you're probably just as confused as I am… [P9].

Further, it has been shown that patients feel more at ease in communicating their pain levels accurately with healthcare professionals who demonstrate genuine empathy and a sincere commitment to their well‐being, ultimately contributing to positive health outcomes [33, 36, 39]. Interestingly, in the current study, participants from various cultural backgrounds empathised with patients, which could have contributed to positive health outcomes in the study location.

It's frustrating at first, but… I feel a little bit more empathetic in that sense, where I'm like, I know I come from a different culture and a different background… And like I just think like, how would I feel if that was my family member? [P9].

The task‐driven, fast‐paced workplace environment, particularly in ED, influenced participants' adaptations to prioritise efficiency, especially when radiographers faced significant workload pressures during busier shifts. Although research literature signifies the importance of individualised, compassionate patient‐centred care [7, 30, 32, 40, 41], participants highlighted substantial challenges tied to patients' presentations, compromised mobility and the severity of language barriers. Further, participants acknowledged the significance of acquiring optimal images and completing examinations, even when faced with perceived barriers.

It's pressuring when you see a patient who speaks a different language because you know that, number one, the patient's probably confused…you want to provide the best, you know, the most diagnostic images and it's just a shame [that] often it's the communication barrier that's limited you from achieving better imaging for the patient. [P4].

Yeah, frustrated… How could they send a patient in that can't communicate without some interpreter for a scan or an x‐ray or like, without any information as well? [P2].

In some situations, these communication barriers can have noticeable implications on the procedural workflow and lead to procedures being postponed or cancelled. Radiographers qualified in CT emphasised the non‐negotiable nature of patients requiring informed consent for contrast‐enhanced CT scans.

We just postpone it until there's like an interpreter or family available… [P8].

…And if they can't give informed consent, again, we can't proceed until they get an interpreter or a doctor to do a consent for them. [P3].

Hence, in the acute clinical setting, patients with linguistic barriers were frequently redirected back to their respective areas within the ED, except in immediate emergencies or resuscitation situations. Further, participants shared experiences that reinforce the substantial consequences caused by communication breakdowns in medical imaging examinations, and they described:

- The influence of linguistic barriers on patients' presenting clinical indications in the ED following clinical triage.
  A lot of the time, yeah, the doctors and nurses will just send them for x‐rays of body parts and only because they've kind of pointed to the area… [P6].
- Radiographers altered capacity to undergo appropriate clinical decision‐making processes.
  … I don't think that it allows for a lot of repeats…it's harder to position the patient accurately if they don't understand what you want, so in a way, you almost allow for a little bit of a greater margin of error… [P5].
- Patients' misinterpretation of instructions leading up to/during the X‐ray examination, causing a greater communication gap between the patient and radiographer.
  It's the changing instructions that I feel like are the biggest reason for repeating…is not being able to explain properly the changing instructions. Like I've had patients that have just put the gown on over the top of their own clothes. [P11].

### 3.3 Enhanced Patient‐Centred Care Through Culturally Responsive Communication

Participants who could speak the same language spoken by the patients expressed the relief and immediate comfort patients felt when someone in the healthcare setting could understand them. Patients felt the urge to provide more information than needed during the examination.

I feel like they're a lot more, like, grateful when they see someone that they can communicate with properly. [P8].

Sometimes they do give me a little bit too much information that I don't need… [P10].

…and then I just talk to them about, and then I feel like there's already a connection if you just ask them. Then you often get on the topic of, oh, where are you from and stuff like that…And they're a lot… yeah, they're really comfortable. [P6].

Trust and rapport engender a profound sense of inclusion and are a compelling illustration of the advantages of integrating culturally congruent healthcare providers into diverse healthcare teams. At an organisational level, provision of leadership for indigenous populations and advocating for the betterment of their communities' health and well‐being is signficant [6]. The recruitment of a healthcare team that reflects the increasing cultural diversity of the population offers several advantages. Beyond addressing and subsiding language barriers, it enhances culturally appropriate and safe patient care within the team through shared knowledge of adopting customs and practices that cater to the cultural distribution of the patient population [6, 42].

In the current study, participants also emphasised the significance of adapting communication strategies in a manner that avoids any condescension or implying that the patient is ‘less intelligent’ simply because a language barrier exists. This insight underscores the importance of preserving the patient's dignity and promoting respectful, patient‐centred care during linguistic and cultural interactions.

…also to not assume that the patient is, um, less intelligent. I think sometimes when you, when there's a language barrier, people speak slower, they speak in a different tone, you know?…and I just think, you know, the patient is not less intelligent, they just don't understand your language. [P4].

Recognition and respect for diverse cultural backgrounds, beliefs, and values of patients could enhance the quality of care provided. In the current study, male participants offered valuable insights on the impact of gender in the presence of cultural barriers in patient encounters and the extra care taken to respect female patients' cultural and religious needs to ensure patient comfort.

They have a head scarf and they have specifically asked for a female radiographer… because they do not want to take off the scarf… It's a cultural thing and I totally understand that… [P10].

Previous studies demonstrated heightened apprehension exhibited by female patients when cared for by male healthcare professionals, particularly during sensitive imaging procedures involving areas like the breasts or pelvic region, and this apprehension may be attributed to religious or faith‐based needs or personal preference [14, 42, 43, 44]. For Muslim women, religion is the most influential factor in refusing care from an opposite‐sex radiographer regardless of the time‐sensitive nature of ED, especially in circumstances where patients needed to remove articles of clothing, such as a niqab or hijab. However, Islamic values become less restrictive in life‐threatening emergencies [14, 43].

### 3.4 Tailored Communication Methods Based on Contextual Patient Factors

At the core of radiographers practice lies the paramount importance of individualised patient care and implementing communication strategies tailored to contextual patient factors [13, 30, 40, 45]. However, the study participants perceived that, in certain situations, adopting a more efficient ‘in‐and‐out’ approach was advantageous, particularly when conducting examinations on patients with limited mobility or those who were unwell in the emergency department or among hospitalised patients. The primary goal of this approach was to mitigate any discomfort experienced by these patients during the imaging procedure. Participants highlighted the following as what contributes to the difference in communication:

- Patients' perceived mobility status.

ED, I tend to be overly cautious… They don't necessarily have a full medical explanation that's given to you… [P11].

I actually just assume that they're mobile and can move and everything… You find that they're more like, mentally okay. [P11].

b The inclusion of family members or an interpreter with the capacity to translate.
  I feel like generally when someone comes in out at MSC [outpatient] and they don't speak English normally, they come with a family member. So normally there's someone to translate. [P5].

However, this approach also presented significant limitations as participants pointed out that family members could become emotionally involved in urgent imaging situations, potentially affecting their objectivity. Additionally, there were concerns about the accuracy of relaying sensitive information when family members acted as ‘*ad‐hoc*’ interpreters when no certified interpreters or multilingual staff members were available.

Sometimes it does just make the entire procedure just a bit like messy and chaotic because the family members are sometimes like overbearing… But for the most part, I think family members are great and incredibly patient. [P4].

Further, using family members as ‘ad‐hoc’ interpreters has been shown to be less reliable due to their emotional involvement with the patient and the potential to relay sensitive or complex information inaccurately [1, 11, 42]. Contestably, these studies overlook the complexities of the hospital environment from the perspectives of indigenous (Māori) and ethnic minorities in a healthcare system primarily influenced by Westernised culture [35, 36].

c Patients' familiarity with the examination workflow, for example when they have encountered the imaging process previously.

They've already had their initials in ED to then come as like an inpatient, and then they're an outpatient now… Even if you have a language barrier, at least it's already been introduced to you. [P9].

Several studies mention the general lack of accessibility to professional interpretation services in the hospital [10, 11, 42, 46]. In the current study, participants highly valued effective communication facilitated by a diverse MI team, particularly in the general X‐ray department where professional interpreters are not routinely available. Participants were more inclined to ask their multilingual colleagues for interpretation since they were already familiar with the terminology and logistics of the examination, such as confirming patient and referral form details.

If you've got someone like students or, um, qualifieds that speak the same language as the patient, it always helps to get them in. Because they know what they're explaining in terms of like radiography stuff. And then you don't have to wait around for the hospital interpreter. [P2].

We don't have like a translator readily available for all the diverse, like languages we cater to… And it's, we're lucky that we even have a diverse like, um, team that can speak and they can help us in that sense. [P9].

While multilingual staff contributed to the completion of efficient imaging examinations, it is crucial to consider the potential challenges that could include increased workload for multilingual radiographers, disruptions to departmental workflow, and increased pressure to interpret fluently and accurately [9, 11].

### 3.5 Adaptive Communication Strategies

Participants kept meaningful patient interactions at the forefront of their practice. For example, where technological aids (such as Google Translate) were deemed helpful in more profound communication gaps, participants appreciated the seamlessness of incorporating acquired non‐verbal cues and visual aids into their examination workflow to convey the same message. This gave them more control over determining what the patient understood, and the demonstrative, abstract nature of general X‐ray examination instructions made it easier to physically show the patient what they needed to do.

… it's helpful for, like, explaining what's going on in the moment, but then like, it's quite limited because you can only hold up, you know, your phone in front of them for so long. [P8].

… I haven't done that before. Um, so I find that, using translation like a translator app that does takes up quite a lot of time… And most of the time you can, you can demonstrate what you need them to do. [P10].

Language barriers gets very difficult. I don't trust Google Translate in the slightest. When you start to get to things that are a bit more complicated, it gets really harsh. [P11].

Participants also expressed the usefulness of machines that feature preset voice recordings in common languages spoken by the hospital's diverse patient population. Such machines automatically provided specific instructions (e.g., breathing) in the patient's preferred language, significantly streamlining the examination workflow and ensuring optimal work quality.

Lots of our new CT machines have like built in, you know, breathing or well breathing instructions in different languages… [P4].

In addition, some studies have explored using visual aids in their respective departments to assess patient comprehension and (a) observed a substantial decrease in reject rates after introducing pre‐recorded visual instructions accompanied by demonstrative images of examination procedures [47], (b) reported a high response rate from families following the implementation of graphic communication tools, strengthening the connection between healthcare teams and the communities they serve [48]. In the current study, participants exercised their use of non‐verbal communication strategies and visual aids in the presence of linguistic barriers to bridge the patient–radiographer communication gap.

So I would just use my hands. I would just tell them to watch me and then I would demonstrate what I will do. It took like twice as long as normal, but that got me diagnostic images regardless. [P9].

He was here for a chest x‐ray, so I kind of tapped my chest. Um, and like kind of coughed and looked at him in a questioning way and he nodded his head… [P5].

### 3.6 Limitations

This study was a single site study and did not include all the views and opinions of all radiographers in Auckland. However, the sample size reflected the profile of the radiographers within the study location with regard to age, experience and ethnicity background. Further research would be useful to confirm whether these results reflect opinions for other Auckland Radiology Departments, and whether the experience is different in the private sector.

## 4 Conclusion

The experiences shared by qualified radiographers in this descriptive, phenomenological qualitative study underscore the vital role of adaptive communication strategies and demonstrative techniques in addressing the challenges associated with linguistic and cultural barriers within the radiology department. These experiences illuminate the intricacies of patient interactions within a multicultural environment, emphasising the importance of healthcare professionals' dedication to culturally safe practice to foster the provision of patient‐centred care. This adaptability enhances mutual understanding between radiographers and patients and contributes to more favourable health outcomes.

## Conflicts of Interest

The authors declare no conflicts of interest.

## Data Availability

The data that support the findings of this study are available from the corresponding author upon reasonable request.
